# Performing Routine Appendectomy in Mucinous Borderline Ovarian Tumours: An Experience of 15 Years From a Cancer Centre

**DOI:** 10.7759/cureus.65580

**Published:** 2024-07-28

**Authors:** Maria Habib, Kheyal Azam Khalil, Zain Tayyab, Afshan Saeed Usmani, Yashfeen Ahmed, Ansab Shah, Aamir Ali Syed

**Affiliations:** 1 Surgical Oncology, Shaukat Khanum Memorial Cancer Hospital and Research Centre, Lahore, PAK

**Keywords:** mucinous tumours, malignancy, appendectomy, ovarian, borderline

## Abstract

Background: It is very controversial whether appendectomy should be performed as a routine part of the staging procedure for mucinous borderline ovarian tumours (mBOTs) or not, as the involvement of the appendix in women undergoing surgery for mBOTs and the exact magnitude of the benefit of appendectomy are unclear. This study was conducted to determine the frequency of appendiceal involvement in patients of mBOTs and morbidity associated with surgery and to evaluate recurrence-free survival after surgery.

Methods: This retrospective cohort study was conducted at a cancer centre from January 2008 to December 2022 (15 years). The hospital database was searched for patients whose final diagnosis was mBOTs. All women who have been operated for mBOTs were included in the study. Descriptive analysis was performed for study variables. The survival curve was calculated according to the Kaplan-Meier method.

Results: Ninety cases whose final diagnosis was mBOTs were identified from the Cancer Registry. Of those, 39 cases were excluded as they did not fulfil the inclusion criteria. Fifty-one patients were finally selected for the study. Of the 51 patients, appendectomy was not performed in eight patients, and the reason was not mentioned in the hospital record. The appendix was macroscopically abnormal in only two patients. None of the patients was diagnosed with mucinous borderline tumours of the appendix in our study. The appendectomy itself was not associated with any complications. Only one patient with mBOT had recurrence after four months of surgery, which was surgically treated and no patient died during the median follow-up of 36 months.

Conclusion: If the appendix is grossly normal looking, then appendectomy can be omitted in surgery of mBOTs. mBOTs have good recurrence free and overall survival outcomes post surgery.

## Introduction

Borderline ovarian tumours are rare tumours that have various subtypes. Mucinous borderline ovarian tumours (mBOTs) comprise up to half of all BOTs in Europe, but this is the more common type in Asia, comprising up to 70% [1]. Ovarian mBOTs have been re-categorized by the World Health Organization (WHO) to include tumours that exhibit gastrointestinal differentiation. Tumours with an endocervical type have been removed from this category now [2].

mBOTs are differentiated from mucinous adenocarcinoma as they do not invade the stroma, although the invasion of the stroma is very difficult to demonstrate [3]. Recently published data have highlighted that mBOTs have a tendency to recur more as invasive cancer as compared to serous Borderline ovarian tumours. The literature has demonstrated that mBOTs have a recurrence rate of 13% in 10 years even after surgery [4].

The overall prognosis and survival of mBOTs are good as survival rates are more than 90% [5]. mBOTs have a very close association with primary appendiceal tumours because of gastrointestinal differentiation so they can present as a metastatic disease from the appendix. Pseudomyxoma peritonei (PMP) is a condition in which extracellular mucin gets deposited in the peritoneum. Previously, it was thought that PMP occurs because of the spread of a ruptured mucinous tumour of the ovary. However, now, it has been proven that its origin is the appendix. Thus, routine appendectomy has been a part of surgery for mBOTs and invasive mucinous ovarian tumours (IMOTs) for many years as removal of the appendix can help in more accurate diagnosis [6-8].

Therefore, some surgeons prefer the removal of the appendix as an essential part of staging and treatment of mBOTs, since it might be helpful in ruling out a primary appendiceal origin of the ovarian neoplasm. Contrary to this, others believe that there is insufficient evidence to support appendectomy for patients with a grossly normal appendix in mBOT and mucinous ovarian cancers. Song et al. and other studies have highlighted that the frequency of appendiceal involvement in mBOTs is less than 1% in either primary or metastatic appendiceal neoplasms. Thus, intraoperative appearance of the appendix should be the deciding factor whether the appendix has to be removed or not, and appendectomy is only warranted when the appendix is grossly abnormal [9,10].

However, it is very controversial whether appendectomy should be performed as a routine part of the staging procedure for mBOTs or not, as the involvement of the appendix in women undergoing surgery for mBOTs and the exact magnitude of the benefit of appendectomy is unclear. Thus, this study has been conducted to assess how often the appendix is involved in patients who undergo surgery for mBOTs and to determine associated morbidity and recurrence of mBOTs after surgery.

## Materials and methods

After obtaining approval from the Institutional Review Board of Shaukat Khanum Memorial Trust, a retrospective cohort study was performed in Shaukat Khanum Memorial Cancer Hospital and Research Centre, Lahore, Pakistan. Data were collected from the hospital cancer registry. Patients who underwent surgery for adnexal mass in our hospital between January 2008 and December 2022 (15 years) and whose final diagnosis was mBOTs were included in the study. Patients whose surgery was not performed in our hospital, who have had prior appendectomy, and those diagnosed with mucinous ovarian cancer were excluded from the study.

The type of surgery, whether laparotomy or laparoscopy, was decided by the preference of the surgeon. The decision of fertility-preserving surgery (unilateral salpingo-oophorectomy with conservation of the contralateral ovary, omentectomy, and multiple biopsies) or completion surgery (hysterectomy with bilateral salpingo-oophorectomy, omentectomy, and multiple biopsies) was based on the patient’s age and desire to preserve fertility and intraoperative findings. Demographic details of the patients included age, parity, preoperative CA-125, and colonoscopy findings. Surgery details included fertility-preserving surgery or completion surgery, the gross appearance of the appendix, appendectomy performed or not, operative time (in minutes), and intraoperative blood loss (in ml). The appendix was classified as normal if it is normal in size, pinkish white in colour, smooth texture, no abnormalities of shape, and absence of any other suspicious-looking features. However, if it does not fulfil these criteria, then it is classified as an abnormal-looking cervix. Postoperative details included postoperative hospital stay (in days) and postoperative complications. Histopathological FIGO staging of the disease was noted. Recurrence-free survival and overall survival were also recorded.

Data were analysed using IBM SPSS Statistics for Windows, Version 23.0 (released 2015, IBM Corp., Armonk, NY). Data were presented as mean ± standard deviation (SD) or median (interquartile range, IQR) for quantitative variables and frequency (%) for qualitative variables. Recurrence-free survival and overall survival were calculated by the Kaplan-Meier method. Recurrence-free survival is defined as the period from surgery to the recurrence of the tumour or the last patient contact. The overall survival is defined as the period from surgery to death or the last patient contact.

## Results

Ninety cases were identified from the Cancer Registry whose final diagnosis was mBOTs. Of those, 39 cases were excluded as they did not fulfil the inclusion criteria. Fifty-one patients were finally selected for the study. Table 1 explains the baseline characteristics of the study participants.

**Table 1:** Baseline characteristics of the study participants SD: standard deviation, FIGO: The International Federation of Gynaecology and Obstetrics

Variables	N (51)
Mean age in years (SD)	36.1 (14.0)
Mean preoperative CA-125 U/ml (range)	92.6 (3-3245)
Colonoscopic findings	N (%)
1. Normal	42 (82.4)
2. Abnormal	0
3. Not performed	09 (17.6)
Approach	N (%)
1. Open	31 (60.8)
2. Laparoscopic	20 (39.2)
Type of surgery	N (%)
1. Completion surgery	27 (52.9)
2. Fertility sparing surgery	24 (47.1)
FIGO Stage	N (%)
Stage 1	
1a	48 (94.1)
1b	0
1c	03 (5.9)

**Figure 1:**
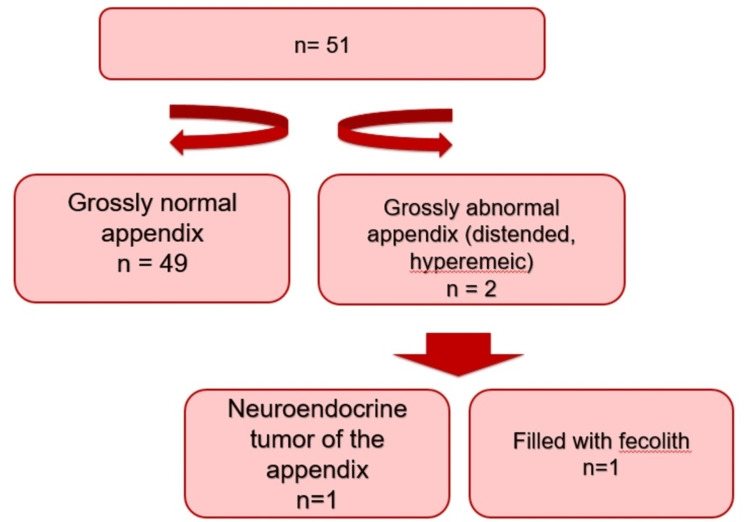
Gross appearance and histopathology of the appendix

Of 51 patients, appendectomy was not performed in eight patients, and the reason was not mentioned in the hospital record. The appendix was normal in imaging findings in all of the cases. Only two patients had a macroscopically abnormal-looking appendix. None of the patients were diagnosed with mBOT of the appendix, as shown in Figure 1.

As described in Table 2, only one patient (2%) had a bladder injury that was diagnosed intraoperatively and repaired during primary surgery. Two patients (3.9%) had delayed postoperative complications in the form of hernia formation. None of the patients had any complications directly related to the appendectomy itself.

**Table 2:** Morbidity associated with surgery SD: standard deviation

Variables	N (51)
Mean operative time in minutes (SD)	115.0 (52.1)
Mean blood loss in ml (SD)	75.8 (68.4)
Mean hospital stay in days (SD)	4.4 (1.7)
Complications	N (%)
1. Intraoperative (bladder injury)	1 (2)
2. Postoperative (hernia formation)	2 (3.9)

**Figure 2:**
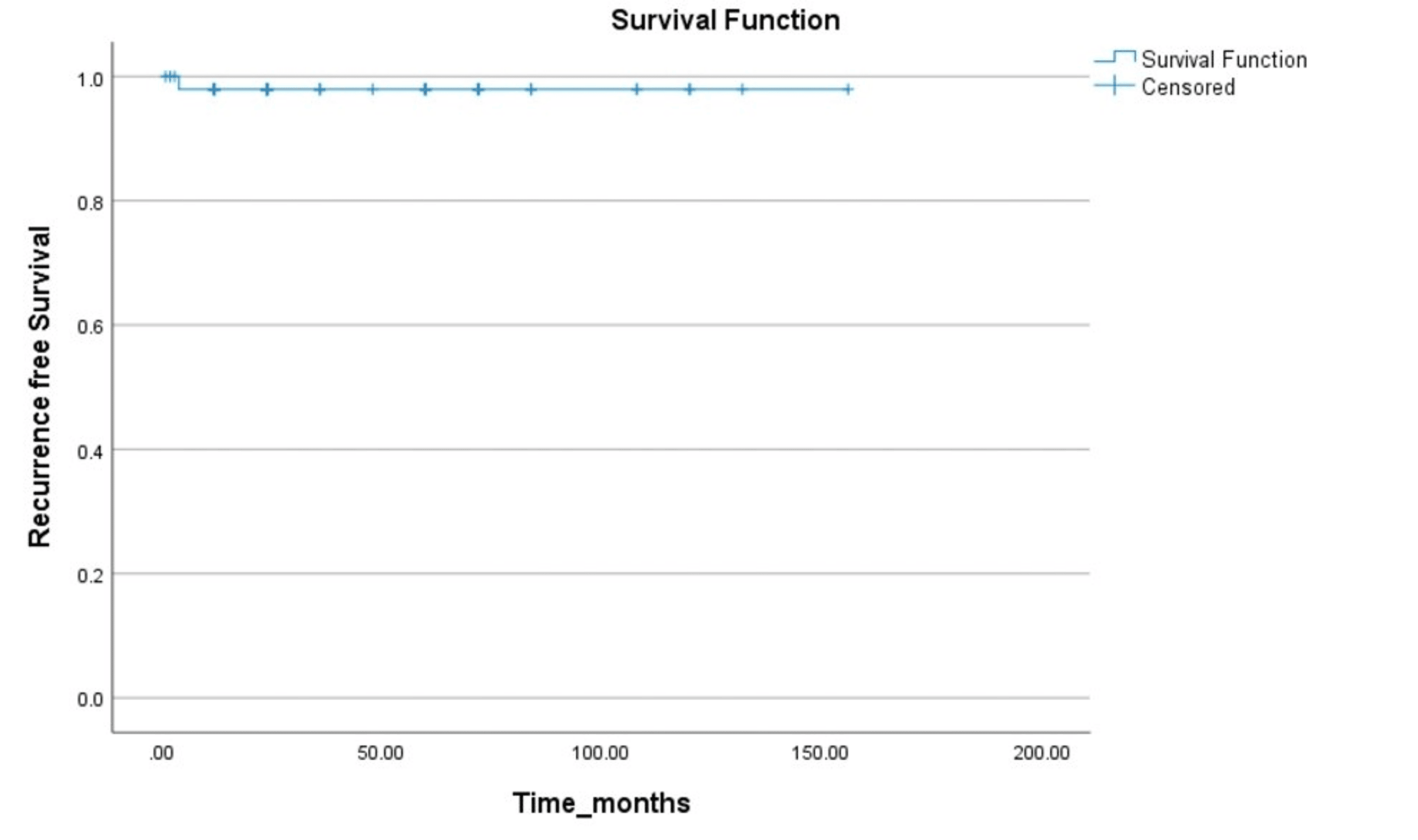
Recurrence-free survival

Figure 2 summarizes the recurrence-free interval of the patients with mBOTs. All patients were followed up during a median time period of 36 months. Only one patient (2%) had recurrence after four months of primary surgery in the form of pelvic mass involving the sigmoid colon, which too was surgically managed and belonged to a group in which appendectomy was performed. Histopathology had shown that the tumour was invading the mesentery of the sigmoid colon. All patients (100% ) were alive during a median follow-up of 32 months, significantly highlighting the 100% overall survival.

## Discussion

The small number of patients (n = 51) over a period of 15 years in our dedicated cancer centre represents the rarity of mBOTs. Our study has highlighted that there are very less chances of the involvement of the appendix in the cases of mBOTs. None of the patients in our study had mBOT metastasizing from the appendix. Although appendectomy itself is associated with minimal complications, it seems to be an unnecessary procedure if the appendix is grossly normal-looking [11].

Cosyns et al. performed a systematic review, and out of 246 articles, 12 articles from different countries (to include diversity) were selected for the final review. The primary outcome was to assess the involvement of the appendix in mBOTs. It was highlighted that the appendix was involved in only 0.86% of the cases. One finding was strikingly common in all those studies that there was no appendiceal involvement in a normal-looking appendix, signifying that there are very less chances of microscopic involvement of the appendix with the tumour. These findings are comparable to the results of our study [11]. Kleppe et al. included patients with primary mucinous appendiceal tumours along with mBOTs in their study and the appendix was macroscopically abnormal-looking in all cases of primary mucinous appendiceal tumours. However, when the appendix was normal-looking, there were no microscopic deposits of the tumour, again highlighting that there is no need for routine appendectomy in a normal-looking appendix [12]. Babaier et al. after a careful review of the literature also mentioned that there were no cases of mBOTs in the literature when the appendix was normal-looking and it would be involved with disease [13].

Rosendahl and colleagues evaluated cases of mucinous adenocarcinoma from the Danish cancer database and found 269 mucinous adenocarcinomas in the ovary. Out of these, 172 underwent appendectomy, and histopathology revealed that 10 cases had metastases of the tumour to the appendix. Of these 10 cases, only two patients had normal-looking appendices, highlighting the microscopic involvement of the appendix in cases of mucinous adenocarcinoma even when it was grossly normal-looking. However, these findings were limited to mucinous adenocarcinoma and cannot be implemented in cases of mBOTs [14]. Similarly, there are few case reports where appendiceal tumours presented as bilateral ovarian masses as their first presentation, but again, the intraoperative appearance of the appendix is of crucial importance even when the patients are being operated for undiagnosed ovarian masses as ovarian masses could be secondary to metastatic disease from the appendix [15].

In contrast to our results, Lavecchia and colleagues performed a retrospective review of 225 cases with mucinous ovarian neoplasms to evaluate if intraoperative factors like the frozen section of the appendix or the surgeon’s impression of appendiceal pathology can be used as a guide for appendectomy. Their final conclusion was that appendectomy along with the removal of mesoappendix should be considered in all cases of mucinous ovarian neoplasms because intraoperative factors showed a negative predictive value of 85.1%, and the remaining patients were misclassified [16].

Our study showed that there were no appendectomy-related complications, which is comparable to the results of other studies. A systematic review done by Manuel and colleagues found that there was no higher complication rate in patients who underwent appendectomy. It highlights that although there is no harm in performing appendectomy in cases of mucinous borderline tumours because of its low complication rate, it can be avoided if the appendix is grossly normal-looking. The most important factor could be the preference of surgeons and how confident they feel to avoid unnecessary procedures in normal intraoperative findings [17].

There was only one patient in our study who had a recurrence after four months of primary surgery that was surgically managed, and none of the patients died over a median follow-up of 36 months, highlighting good survival outcomes. Sevki Göksun Gökulu and colleagues included 80 cases of mBOTs and they demonstrated that appendectomy or hysterectomy did not improve recurrence-free or overall survival in cases of mBOTs. Similarly, other studies have also highlighted that there were no statistically significant improved survival outcomes whether appendectomy was performed or not. Our study showed 100% overall survival, which is in line with the current literature that borderline ovarian tumours have a very good recurrence-free and overall survival approaching 100% [17,18].

The limitations of our study were the retrospective design and the small sample size of the study population because of the rarity of the tumour. However, as the data were retrieved from the hospital database, it is most likely to be accurate. To generalize the results, this study can be conducted in multicentres and the outcomes can lead to changing practices while managing mBOTs.

## Conclusions

Microscopic involvement of a appendix in grossly normal looking appendix is extremely rare. Although appendectomy is not associated with any major complications, if the appendix is grossly normal-looking, then appendectomy can be omitted in surgery of mBOTs. mBOTs have good recurrence-free and overall survival outcomes post surgery. More prospective multicenter trials should be conducted to generalize the results to change current practices while managing these tumors.
